# Sex-related disparities in the incidence and outcomes of infective endocarditis according to type 2 diabetes mellitus status in Spain, 2016–2020

**DOI:** 10.1186/s12933-022-01633-2

**Published:** 2022-09-30

**Authors:** Ana Lopez-de-Andres, Rodrigo Jimenez-Garcia, Valentin Hernández-Barrera, Javier de-Miguel-Díez, Jose M. de-Miguel-Yanes, David Martinez-Hernandez, David Carabantes-Alarcon, Jose J. Zamorano-Leon, Concepción Noriega

**Affiliations:** 1 Department of Public Health & Maternal and Child Health, Faculty of Medicine, Universidad Complutense de Madrid, IdISSC, 28040 Madrid, Spain; 2 Preventive Medicine and Public Health Teaching and Research Unit, Health Sciences Faculty, Rey Juan Carlos University, Alcorcón, Madrid, Spain; 3 Respiratory Care Department, Hospital General Universitario Gregorio Marañón, Universidad Complutense de Madrid, Instituto de Investigación Sanitaria Gregorio Marañón (IiSGM), Madrid, Spain; 4 Internal Medicine Department, Hospital General, Universitario Gregorio MarañónUniversidad Complutense de MadridInstituto de Investigación Sanitaria Gregorio Marañón (IiSGM), Madrid, Spain; 5 Department of Nursery and Physiotherapy, Faculty of Medicine and Health Sciences, University of Alcalá, Alcalá de Henares, Spain

**Keywords:** Infective endocarditis, Diabetes, Hospitalization, Mortality, Sex differences

## Abstract

**Background:**

We performed a study to assess sex-differences in incidence (2016–2020), clinical characteristics, use of therapeutic procedures, and in-hospital outcomes in patients with infective endocarditis (IE) according to T2DM status.

**Methods:**

Ours was a retrospective cohort study using data from the Spanish National Hospital Discharge Database. We estimated the incidence of hospitalizations for IE in men and women aged ≥ 40 years with and without T2DM. Propensity score matching (PSM) and multivariable logistic regression were used to compare subgroups according to sex and the presence of T2DM.

**Results:**

From 2016 to 2020, IE was coded in 9,958 patients (66.79% men). T2DM was diagnosed in 2,668 (26.79%). The incidence of IE increased significantly from 15.29 cases per 100,000 persons with T2DM in 2016 to 17.69 in 2020 (p < 0.001). However, this increment was significant only among men with T2DM (19.47 cases per 100,000 in 2016 vs. 22.84 in 2020; p = 0.003). The age-adjusted incidence of IE was significantly higher in people with T2DM (both sexes) than in those without T2DM (IRR, 2.86; 95% CI, 2.74–2.99). The incidence of IE was higher in men with T2DM than in women with T2DM (adjusted IRR, 1.85; 95% CI, 1.54–3.31). After PSM, in-hospital mortality (IHM) was higher among T2DM women than matched T2DM men (22.65% vs. 18.0%; p = 0.018).

The presence of T2DM was not associated with IHM in men or women.

**Conclusions:**

T2DM is associated with a higher incidence of hospitalization for IE. Findings for T2DM patients who had experienced IE differed by sex, with higher incidence rates and lower IHM in men than in women. T2DM was not associated to IHM in IE in men or in women.

**Supplementary Information:**

The online version contains supplementary material available at 10.1186/s12933-022-01633-2.

## Background

People with type 2 diabetes mellitus (T2DM) are at greater risk of infection and experience worse outcomes of infection than those without diabetes [1]. Infective endocarditis (IE) is an infection whose incidence has increased in recent years [2–4] and that is highly influenced by age and the presence of comorbidities, such as diabetes [5]. In Denmark, a study based on nationwide registries found that the incidence of IE increased from 5.0/100,000 per year in 1997 to 10.5/100,000 per year in 2017 [6]. The incidence of IE in patients with T2DM is also increasing [7]. A cohort study based on the Spanish population reported a significant increase in incidence among patients with T2DM between 2001 and 2015 (from 6.0/100,000 to 13.1/100,000 per year; p < 0.001). Furthermore, incidence was significantly higher in people with T2DM than in those without diabetes (incidence rate ratio [IRR], 2.2; 95% confidence interval [CI], 2.1–2.3) [8].

The prognosis for IE in the general population is poor, with an in-hospital mortality (IHM) of around 25% [9]. In Spain, approximately 20.8% of patients with T2DM admitted to hospital with IE die in hospital [8]. Several studies have found diabetes to be an independent predictor of IHM [5, 10, 11]. The study by Tahon et al. [11] (inclusion period of 7 years) revealed that patients with diabetes have a higher risk of dying after IE (hazard ratio [HR] 2.24; 95% CI, 1.46–3.45).

The incidence and outcome of IE may differ according to sex. Hospital-based studies show a higher incidence of IE in men and a male-to-female ratio ranging from 1.3:1 to 3:1 [12, 13]. Furthermore, several studies indicate that female sex was independently associated with mortality [14, 15]. Based on data from a multicenter cohort in Spain between 2008 and 2018, Varela Barca et al. [16] found that the OR for mortality in females was 1.41; (95% CI, 1.21 to 1.65; p < 0.001). Reported results for patients with diabetes are like those for the general population [8, 17]. In contrast, other studies report a trend toward lower IHM among women [18]. Further confusing the picture, Sevilla et al. [19], reported nonsignificant differences in IHM between both sexes. However, in this study, IHM was 28% among men vs. 35% among women (p = 0.1), thus indicating that lack of statistical power due to small populations may generate misleading conclusions [19].

Recent studies have concluded that left ventricle is subject to maladaptive changes involving left ventricular mass and myocardial mechanical energetic efficiency especially in women with newly diagnosed T2DM. This sex-differences may contribute to explain, at least in part, the stronger impact of T2DM on the excess risk of cardiovascular disease in women than in men [20].

Using a national administrative database, we compared incidence, clinical characteristics, use of therapeutic procedures, and in-hospital outcomes among patients with IE according to T2DM status and sex over a 5-year period. Propensity score matching (PSM) was used to compare outcomes between men and women with and without T2DM and between men and women with T2DM. Finally, we identified the variables independently associated with IHM for patients with T2DM and IE according to sex.

## Methods

### Study design, study population, and data assessment

Our cohort study was based on hospital discharge reports collected through the Hospital Discharge Records of the Spanish National Health System (RAE-CMBD, *Registro de Actividad de Atención Especializada-Conjunto Mínimo Básico de Datos* (RAE-CMBD) [Register of Specialized Care–Basic Minimum Database]). The study period covered 1 January 2016 to 31 December 2020. The discharge records are coded based on the International Classification of Disease, Tenth Revision (ICD-10). The RAE-CMBD was implemented in Spain in year 1987 and the methodology has remained basically unchanged overtime. Details on the RAE-CMBD are available online [21]. The total number of hospital discharges of people aged 40 year or over in Spain recorded in the RAE-CMBD from 2016 to 2020 were 16,356,876. The distribution according to year, and sex can be found in Additional file 1: Table S1. As can be seen in the table men outnumbered women in all the years studied, representing 52.36% of hospital discharges from 2016 to 2020.

The study population comprised patients aged ≥ 40 years with a diagnosis of IE (ICD-10 codes: I33.0; I33.9; I38) in primary or secondary diagnostic position registered in the discharge records, as described by Olmos et al. [3]. Only the first episode was recorded in cases where the same patient was hospitalized more than once during the study period.

We stratified the study population according to sex and to the presence of T2DM. Patients with a diagnosis code for T2DM (E11.x) in any field were classified as having T2DM. Patients with a code for type 1 diabetes mellitus (E10.x) in any field were excluded.

In the RAE-CMBD are codified all the clinical conditions the patients have when admitted the hospital and those conditions that are not present on admission which are detected during the hospitalization [21]. So, patients with T2DM will have a code for this condition beside if they have been previously diagnosed and treated for T2DM in the hospital where the IE hospitalization takes place, in any other hospital or at primary care centers. If fact, in Spain most T2DM patients are diagnosed, treated, and controlled at primary care.

The main study variables were trends in the incidence of IE in patients with and without T2DM, IHM, and length of hospital stay (LOHS). We also analyzed comorbidities and therapeutic procedures in patients with IE.

Incidence rates of hospitalization for IE in persons with and without T2DM were calculated by age group and sex using data from the Spanish Institute of Statistics and applying the methods described by de Miguel-Yanes et al. [8, 22].

Comorbidity was quantified using the Charlson Comorbidity Index (CCI), which calculated based on ICD-10 codes, excluding diabetes, as described elsewhere [23, 24]. Specifically, we reported results for the following diagnoses: atrial fibrillation, COVID-19, ischemic heart disease, periannular complications/atrioventricular block, previous valve disease (aortic, mitral, tricuspid, and pulmonary), septic arterial embolism, and cardiogenic shock. Furthermore, we retrieved data on prosthetic valve carriers.

Using ICD-10 codes, we collected data on the following in-hospital procedures: dialysis, heart valve surgery (aortic, mitral, tricuspid, pulmonary), mechanical ventilation, and pacemaker implantation, irrespective of where they appeared in the procedure coding fields.

As for pathogens in patients with IE, we included *Staphylococcus* bacteremia, *Streptococcus* bacteremia, Gram-negative bacteremia, and fungemia. The ICD-10 codes used to identify these diagnoses and procedures can be found in Additional file 1: Table S2.

PSM was applied to generate subpopulations that were comparable according to their baseline conditions [25]. The three PSM analyses performed were as follows: men with and without T2DM, women with and without T2DM, men with T2DM and women with T2DM. The PSM was based on multivariable logistic regression, in which the matching variables were year of admission, age, sex, and comorbid conditions at admission. These methods have been described in detail elsewhere [8].

### Statistical analysis

We obtained the incidence of hospitalizations for IE per 100,000 persons with and without T2DM for each of the 5 years analyzed. Poisson regression was used to calculate age- and sex-adjusted IRR with their 95% CI.

The descriptive statistical analysis was based on mean and standard deviation (SD) or median and interquartile range (IQR) for continuous variables and frequency and percentage for categorical variables.

Continuous variables were compared using the *t* test or Mann–Whitney test. Categorical variables were compared using the chi-square test.

We conducted Cochran-Armitage tests to assess the time trend from 2016 to 2020 for categorical variables. We used the linear regression T test and Jonckheere-Terpstra test for means and medians, respectively.

Multivariable trends in incidence of IE, adjusted by age and sex as required, were assessed using Poisson regression. We provided the annual percentage change (APC) with 95% confidence interval (CI).

Multivariable logistic regression analysis was applied to identify those variables that were independently associated with IHM. Separate models were constructed for men and women based on T2DM status. Finally, the effect of sex was assessed using the database of people with T2DM. The effect of T2DM was studied using the entire database of patients hospitalized with IE. The results are shown as odds ratios (ORs) with their 95% CIs.

Two-way interactions were examined to identify whether sex modified the effect of T2DM on outcomes. However, none of the statistical test for interaction between sex and T2DM showed a significant result in the multivariable models.

Stata version 14 (Stata, College Station, Texas, USA) was used for the statistical analysis and PSM. A p value < 0.05 (2-sided) was considered significant.

### Ethics

The RAE-CMBD is owned by the Spanish Ministry of Health and can be accessed upon request [26]. According to Spanish legislation, neither individual written consent from the patients nor ethics committee approval is required as this registry is anonymous.

## Results

A total of 9,958 patients (66.79% men and 33.21% women) aged 40 years or over were hospitalized with a diagnosis of IE in Spain during 2016–2020, so the yearly incidence has been 7.60 cases per 100,000 inhabitants’ ≥ 40 years.

T2DM was diagnosed in 2,668 patients (26.79%). T2DM was more prevalent among men than among women (27.50% vs. 25.31%; p < 0.001).

### Incidence of patients admitted to hospital with IE according to T2DM status

In patients with T2DM (men and women), the incidence of IE increased significantly from 15.29 cases per 100,000 persons with T2DM in 2016 to 17.69 in 2020 (p < 0.001). When the analysis was stratified by sex, this increment was significant only in men with T2DM (19.47 cases per 100,000 men with T2DM in 2016 vs. 22.84 in 2020; p = 0.003) (Table 1). Multivariable Poisson regression showed that there was a significant increase in the incidence of IE over time for people with T2DM (Age and sex adjusted APC 1.66%; 95% CI, 1.43% to 1.90%; p < 0.001) and among men with T2DM (Age adjusted APC, 2.82%; 95% CI, 2.17% to 3.48%; p = 0.005).

**Table 1:** Incidence, clinical characteristics and in-hospital outcomes of patients hospitalized with infective endocarditis in Spain from 2016 to 2020 according to T2DM status

		2016	2017	2018	2019	2020	p-value
N, (incidence per 100,000 subjects per year)	T2DM	468 (15.29)	534 (17.45)	538 (18.06)	615 (21.21)	513 (17.69)	< 0.001
	No T2DM	1385 (6.12)	1411 (6.23)	1615 (6.93)	1518 (6.33)	1361 (5.68)	0.111
N, (incidence per 100,000 women per year)	T2DM	154 (10.64)	165 (11.4)	160 (11.36)	196 (14.3)	164 (11.96)	0.056
	No T2DM	458 (3.82)	481 (4.01)	577 (4.69)	490 (3.87)	463 (3.66)	0.385
N, (incidence per 100,000 men per year)	T2DM	314 (19.47)	369 (22.88)	378 (24.07)	419 (27.42)	349 (22.84)	0.003
	No T2DM	927 (8.7)	930 (8.72)	1038 (9.44)	1028 (9.07)	898 (7.92)	0.161
Age, mean (SD)	T2DM	72.94 (10.09)	72.5 (9.62)	72.78 (9.62)	72.53 (9.99)	73.66 (9.58)	0.298
	No T2DM	69.76 (13.23)	69.26 (13.19)	70.16 (13.54)	69.47 (13.23)	69.99 (12.7)	0.330
CCI index, mean (SD)	T2DM	1.26 (1.07)	1.4 (1)	1.5 (1.13)	1.5 (1.1)	1.56 (1.11)	< 0.001
	No T2DM	1.06 (1.04)	1.16 (1.05)	1.15 (1.04)	1.1 (1.03)	1.2 (1.08)	0.006
Previous aortic valve disease, n (%)	T2DM	115 (24.57)	146 (27.34)	118 (21.93)	175 (28.46)	135 (26.32)	0.109
	No T2DM	356 (25.7)	412 (29.2)	469 (29.04)	446 (29.38)	455 (33.43)	0.001
Previous mitral valve disease, n (%)	T2DM	110 (23.5)	121 (22.66)	145 (26.95)	156 (25.37)	138 (26.9)	0.378
	No T2DM	381 (27.51)	396 (28.07)	457 (28.3)	432 (28.46)	430 (31.59)	0.138
Previous pulmonic valve disease, n (%)	T2DM	0 (0)	3 (0.56)	0 (0)	1 (0.16)	0 (0)	0.080
	No T2DM	4 (0.29)	5 (0.35)	3 (0.19)	1 (0.07)	5 (0.37)	0.430
Previous tricuspid valve disease, n (%)	T2DM	24 (5.13)	28 (5.24)	44 (8.18)	60 (9.76)	42 (8.19)	0.011
	No T2DM	74 (5.34)	97 (6.87)	138 (8.54)	126 (8.3)	140 (10.29)	< 0.001
Prosthetic valve carriers, n (%)	T2DM	59 (12.61)	44 (8.24)	57 (10.59)	53 (8.62)	43 (8.38)	0.085
	No T2DM	132 (9.53)	140 (9.92)	144 (8.92)	123 (8.1)	128 (9.4)	0.483
LOHS, Median (IQR)	T2DM	19 (27)	17 (27)	18.5 (24)	19 (25)	18 (23)	0.633
	No T2DM	19 (24)	19 (27)	19 (27)	19 (24)	19 (23)	0.994
IHM, n (%)	T2DM	89 (19.02)	104 (19.48)	93 (17.29)	104 (16.91)	102 (19.88)	0.622
	No T2DM	226 (16.32)	207 (14.67)	280 (17.34)	276 (18.18)	249 (18.3)	0.058

T2DM: Type 2 diabetes mellitus; CCI: Charlson comorbidity index; LOHS: Length of hospital stay; IHM: in-hospital mortality

In women with T2DM and in patients without T2DM (both sex, men and women separately), the incidence of admissions remained stable over the study period after bivariate and multivariate analysis.

The incidence of IE was significantly higher in people with T2DM than in people without for all the years analyzed (p < 0.001). The age- and sex-adjusted IRR was 2.86 (95% CI, 2.74–2.99; Poisson regression) for the incidence of IE in people with T2DM compared with those without.

Over time, the CCI increased significantly in patients with and without T2DM (p < 0.001 and p = 0.006). Furthermore, the presence of previous tricuspid valve disease increased significantly (p = 0.011 in patients with T2DM and p < 0.001 in patients without T2DM). The presence of previous aortic valve disease increase significantly only in patients without T2DM (25.7% in 2016 vs 33.43% in 2020; p = 0.001) (Table 1).

LOHS was around 19 days for all the years analyzed and diabetes status, and IHM remained stable in patients with T2DM (19.02% in 2016 vs. 19.88% in 2020; p = 0.622) and in patients without T2DM (16.32% in 2016 vs. 18.3%; p = 0.058) (Table 1).

**Fig. 1:**
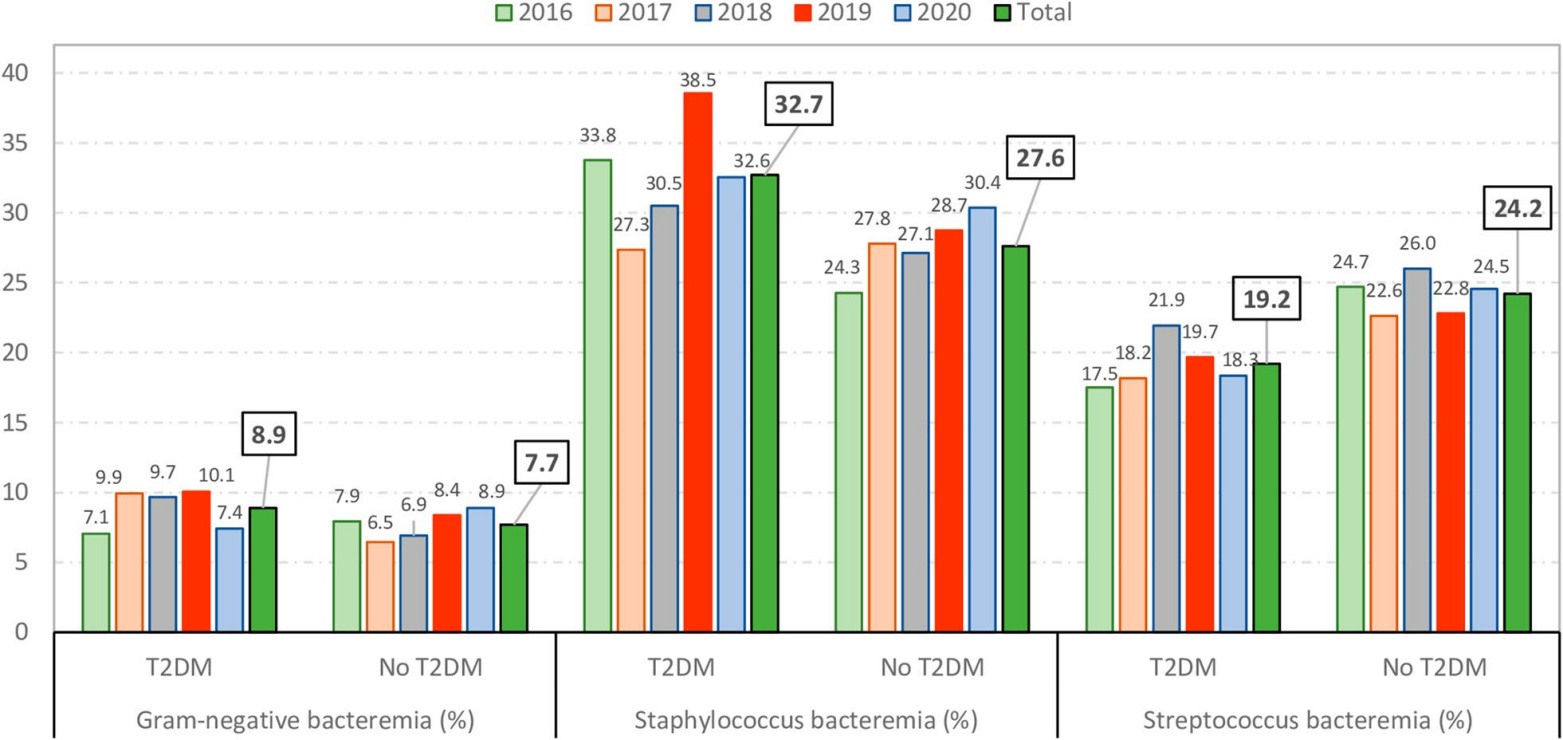
Distribution of more frequent pathogens in patients with and without T2DM with infective endocarditis in Spain (2016–2020)

As can be seen in Fig. 1, for the period 2016–2020, the prevalence of *Staphylococcus* bacteremia (32.7% vs. 27.6%; p < 0.001) and Gram-negative bacteremia (8.9% vs. 7.7%; p = 0.051) were higher among patients with T2DM whereas, *Streptococcus* bacteremia was more frequent among those without T2DM (24.2% vs. 19.2%; p < 0.001).

Over time, the distribution of pathogens in IE patients with and without T2DM remained stable, although we found that *Staphylococcus* bacteremia decreased significantly in patients with T2DM (p = 0.001) but increased significantly in patients without diabetes (p = 0.007) (Additional file 1: Table S3).

### Admissions to hospital with a diagnosis of IE: Clinical characteristics and hospital outcomes according to T2DM status

The incidence of IE was significantly higher in women with T2DM than in those without T2DM (11.91 cases per 100,000 vs. 4.01 cases per 100,000; p < 0.001) (Table 2). The Poisson regression corresponding age-adjusted IRR obtained was 2.97 (95% CI, 2.75–3.21).

**Table 2:** Distribution of study covariates and hospital outcomes of WOMEN with and without T2DM with infective endocarditis in Spain (2016–2020), before and after propensity score matching (PSM)

	Before PSM			After PSM		
	T2DM	No T2DM	p-value	T2DM	No T2DM	p-value
N, (incidence per 100,000 women per year)	839 (11.91)	2469 (4.01)	< 0.001	839	839	NA
Age, mean (SD)	75.58 (9)	73.13 (12.57)	< 0.001	75.58 (9)	76.81 (10.12)	0.008
40–66 years old, n (%)	122 (14.54)	678 (27.46)	< 0.001	122 (14.54)	122 (14.54)	0.008
67–75 years old, n (%)	264 (31.47)	568 (23.01)		264 (31.47)	209 (24.91)	
≥ 76 years old, n (%)	453 (53.99)	1223 (49.53)		453 (53.99)	508 (60.55)	
Acute renal disease, n (%)	205 (24.43)	489 (19.81)	0.004	205 (24.43)	200 (23.84)	0.775
Chronic renal disease, n (%)	257 (30.63)	394 (15.96)	< 0.001	257 (30.63)	244 (29.08)	0.488
Congestive heart failure, n (%)	146 (17.4)	254 (10.29)	< 0.001	146 (17.4)	129 (15.38)	0.262
COPD, n (%)	35 (4.17)	74 (3)	0.100	35 (4.17)	37 (4.41)	0.810
Dementia, n (%)	34 (4.05)	60 (2.43)	0.015	34 (4.05)	31 (3.69)	0.704
Atrial fibrillation, n (%)	319 (38.02)	872 (35.32)	0.159	319 (38.02)	308 (36.71)	0.579
COVID-19, n (%)	6 (0.72)	13 (0.53)	0.532	6 (0.72)	6 (0.72)	0.999
Ischemic heart disease, n (%)	114 (13.59)	229 (9.28)	< 0.001	114 (13.59)	110 (13.11)	0.774
Previous aortic valve disease, n (%)	206 (24.55)	633 (25.64)	0.533	206 (24.55)	196 (23.36)	0.567
Previous mitral valve disease, n (%)	268 (31.94)	788 (31.92)	0.988	268 (31.94)	263 (31.35)	0.793
Previous pulmonic valve disease, n (%)	2 (0.24)	6 (0.24)	0.981	2 (0.24)	1 (0.12)	0.563
Previous tricuspid valve disease, n (%)	94 (11.2)	233 (9.44)	0.139	94 (11.2)	97 (11.56)	0.818
Prosthetic valve carriers, n (%)	83 (9.89)	241 (9.76)	0.912	83 (9.89)	87 (10.37)	0.746
CCI index, mean (SD)	1.41 (0.99)	1.13 (1)	< 0.001	1.41 (0.99)	1.32 (1.07)	0.097
Periannular complications / atrioventricular block, n (%)	45 (5.36)	93 (3.77)	0.046	45 (5.36)	32 (3.81)	0.129
Septic arterial embolism, n (%)	35 (4.17)	103 (4.17)	0.999	35 (4.17)	23 (2.74)	0.109
Cardiogenic shock, n (%)	14 (1.67)	52 (2.11)	0.434	14 (1.67)	16 (1.91)	0.713
Dialysis, n (%)	64 (7.63)	105 (4.25)	< 0.001	64 (7.63)	33 (3.93)	0.001
Heart valve surgery (aortic, mitral, tricuspid, pulmonary), n (%)	121 (14.42)	414 (16.77)	0.111	121 (14.42)	108 (12.87)	0.355
Mechanical ventilation, n (%)	87 (10.37)	254 (10.29)	0.946	87 (10.37)	74 (8.82)	0.281
Pacemaker implantation, n (%)	41 (4.89)	97 (3.93)	0.231	41 (4.89)	32 (3.81)	0.290
LOHS, Median (IQR)	18 (25)	18 (26)	0.778	18 (25)	18 (26)	0.823
IHM, n (%)	190 (22.65)	498 (20.17)	0.127	190 (22.65)	187 (22.29)	0.861

T2DM: Type 2 diabetes mellitus; CCI: Charlson comorbidity index; COPD: chronic obstructive pulmonary disease; LOHS: Length of hospital stay; IHM: in-hospital mortality

Before PSM, mean age was significantly higher among women with T2DM than in those without (75.58 [SD = 9 years] vs. 73.13 [SD = 12.57 years]), and women with T2DM also had a higher mean CCI (1.41 vs. 1.13: p < 0.001) and more specific chronic conditions, such as acute renal disease, chronic renal disease, congestive heart failure, dementia, ischemic heart disease, and periannular complications/atrioventricular block (Table 2).

During hospitalization, women with T2DM received dialysis significantly more often than women without T2DM (7.63% vs. 4.25%; p < 0.001). The median LOHS was 18 days for women with and without T2DM. The crude IHM was 22.65% for women with T2DM and 20.17% for those without (p = 0.127).

After PSM, dialysis remained more frequent among T2DM women, and IHM was similar, at around 22% in women with and without T2DM (Table 2).

Crude incidence was significantly higher in men with T2DM than in men without T2DM (23.29 cases per 10,000 vs. 8.77 cases per 10,000; p < 0.001). The age-adjusted IRR estimated for men T2DM vs. men without T2DM was 2.65 (95% CI, 2.51–2.80) (Table 3).

**Table 3:** Distribution of study covariates and hospital outcomes of MEN with and without T2DM with infective endocarditis in Spain (2016–2020), before and after propensity score matching (PSM)

	BEFORE PSM			AFTER PSM		
	T2DM	No T2DM	p-value	T2DM	No T2DM	p-value
N, (incidence per 100,000 men per year)	1829 (23.29)	4821 (8.77)	< 0.001	1829	1829	NA
Age, mean (SD)	71.61 (9.88)	67.99 (13.17)	< 0.001	71.61 (9.88)	72.86 (11.07)	< 0.001
40–66 years old, n (%)	543 (29.69)	2066 (42.85)	< 0.001	543 (29.69)	489 (26.74)	< 0.001
67–75 years old, n (%)	590 (32.26)	1147 (23.79)		590 (32.26)	519 (28.38)	
≥ 76 years old, n (%)	696 (38.05)	1608 (33.35)		696 (38.05)	821 (44.89)	
Acute renal disease, n (%)	463 (25.31)	1025 (21.26)	< 0.001	463 (25.31)	452 (24.71)	0.675
Chronic renal disease, n (%)	514 (28.1)	676 (14.02)	< 0.001	514 (28.1)	457 (24.99)	0.033
Congestive heart failure, n (%)	294 (16.07)	432 (8.96)	< 0.001	294 (16.07)	275 (15.04)	0.386
COPD, n (%)	253 (13.83)	448 (9.29)	< 0.001	253 (13.83)	256 (14)	0.886
Dementia, n (%)	31 (1.69)	76 (1.58)	0.732	31 (1.69)	30 (1.64)	0.897
Atrial fibrillation, n (%)	545 (29.8)	1278 (26.51)	0.007	545 (29.8)	564 (30.84)	0.494
COVID-19, n (%)	6 (0.33)	26 (0.54)	0.266	6 (0.33)	3 (0.16)	0.317
Ischemic heart disease, n (%)	506 (27.67)	740 (15.35)	< 0.001	506 (27.67)	477 (26.08)	0.279
Previous aortic valve disease, n (%)	483 (26.41)	1505 (31.22)	< 0.001	483 (26.41)	483 (26.41)	0.999
Previous mitral valve disease, n (%)	402 (21.98)	1308 (27.13)	< 0.001	402 (21.98)	383 (20.94)	0.444
Previous pulmonic valve disease, n (%)	2 (0.11)	12 (0.25)	0.268	2 (0.11)	1 (0.05)	0.564
Previous tricuspid valve disease, n (%)	104 (5.69)	342 (7.09)	0.040	104 (5.69)	88 (4.81)	0.236
Prosthetic valve carriers, n (%)	173 (9.46)	426 (8.84)	0.429	173 (9.46)	163 (8.91)	0.567
CCI index, mean (SD)	1.47 (1.13)	1.14 (1.07)	< 0.001	1.47 (1.13)	1.39 (1.16)	0.053
Periannular complications / atrioventricular block, n (%)	122 (6.67)	288 (5.97)	0.292	122 (6.67)	96 (5.25)	0.069
Septic arterial embolism, n (%)	67 (3.66)	221 (4.58)	0.099	67 (3.66)	52 (2.84)	0.162
Cardiogenic shock, n (%)	35 (1.91)	126 (2.61)	0.097	35 (1.91)	45 (2.46)	0.258
Dialysis, n (%)	118 (6.45)	213 (4.42)	0.001	118 (6.45)	88 (4.81)	0.031
Heart valve surgery (aortic, mitral, tricuspid, pulmonary), n (%)	336 (18.37)	1123 (23.29)	< 0.001	336 (18.37)	320 (17.5)	0.490
Mechanical ventilation, n (%)	207 (11.32)	545 (11.3)	0.988	207 (11.32)	192 (10.5)	0.426
Pacemaker implantation, n (%)	121 (6.62)	244 (5.06)	0.013	121 (6.62)	87 (4.76)	0.015
LOHS, Median (IQR)	19 (25)	20 (25)	0.866	19 (25)	19 (24)	0.556
IHM, n (%)	302 (16.51)	740 (15.35)	0.244	302 (16.51)	333 (18.21)	0.176

T2DM: Type 2 diabetes mellitus; CCI: Charlson comorbidity index; COPD: chronic obstructive pulmonary disease; LOHS: Length of hospital stay; IHM: in-hospital mortality

The differences in the distribution of age and comorbidities between men with and without T2DM were significant before PSM, as in women. However, chronic obstructive pulmonary disease (COPD) was more prevalent among men with T2DM (13.83% vs. 9.29%; p < 0.001) and atrial fibrillation (29.8% vs. 26.51%; p = 0.007). The prevalence of previous valve disease was lower in men with T2DM (Table 3).

As in women, men with T2DM more frequently received dialysis (6.45% vs. 4.42%; p = 0.001). Pacemakers were also more frequent in men with T2DM than in those without T2DM (6.62% vs. 5.06%; p = 0.013), although men with T2DM less frequently underwent heart valve surgery (18.37% vs. 23.29%; p < 0.001). LOHS was around 19 days in both men with and men without T2DM. The crude IHM was 16.51% for men with T2DM and 15.35% for those without (p = 0.244) (Table 3).

After PSM, we found that dialysis and presence of a pacemaker continued to be more frequent in men with T2DM (Table 3).

### Sex-differences in the incidence, clinical characteristics, and hospital outcomes in patients with T2DM admitted to hospital with a diagnosis of IE

Incidence of IE was significantly higher in men with T2DM than in women with T2DM. The overall incidence of hospitalizations for IE over the period 2016–2020 was 1.85 times higher among men with T2DM than among women with T2DM (age-adjusted IRR, 1.85; 95% CI, 1.54–3.31; Poisson regression).

When we compared men and women with T2DM, we observed that men were younger (71.61 ± 9.88 years vs. 75.58 ± 9 years; p < 0.001) and that women had higher prevalence of dementia (4.05% vs. 1.69%; p < 0.001), atrial fibrillation (38.02% vs. 29.8%; p < 0.001), previous mitral valve disease (31.94% vs. 21.98%; p < 0.001), and previous tricuspid valve disease (11.2% vs. 5.69%; p < 0.001). However, COPD and atrial fibrillation were more prevalent in men with T2DM (Table 4).

**Table 4:** Distribution of study covariates and hospital outcomes of MEN AND WOMEN with T2DM with infective endocarditis in Spain (2016–2020), before and after propensity score matching (PSM)

	BEFORE PSM			AFTER PSM		
	T2DM Men	T2DM Women	p-value	T2DM Men	T2DM Women	p-value
N, (incidence per 100,000 subjects per year)	1829 (23.29)	839 (11.91)	< 0.001	839	839	NA
Age, mean (SD)	71.61 (9.88)	75.58 (9)	< 0.001	76.29 (7.65)	75.58 (9)	0.081
40–66 years old, n (%)	543 (29.69)	122 (14.54)	< 0.001	81 (9.65)	122 (14.54)	0.004
67–75 years old, n (%)	590 (32.26)	264 (31.47)		302 (36)	264 (31.47)	
≥ 76 years old, n (%)	696 (38.05)	453 (53.99)		456 (54.35)	453 (53.99)	
Acute renal disease, n (%)	463 (25.31)	205 (24.43)	0.626	200 (23.84)	205 (24.43)	0.775
Chronic renal disease, n (%)	514 (28.1)	257 (30.63)	0.181	247 (29.44)	257 (30.63)	0.594
Congestive heart failure, n (%)	294 (16.07)	146 (17.4)	0.391	133 (15.85)	146 (17.4)	0.394
COPD, n (%)	253 (13.83)	35 (4.17)	< 0.001	40 (4.78)	35 (4.17)	0.554
Dementia, n (%)	31 (1.69)	34 (4.05)	< 0.001	25 (2.98)	34 (4.05)	0.233
Atrial fibrillation, n (%)	545 (29.8)	319 (38.02)	< 0.001	309 (36.83)	319 (38.02)	0.614
COVID-19, n (%)	6 (0.33)	6 (0.72)	0.165	6 (0.72)	6 (0.72)	0.999
Ischemic heart disease, n (%)	506 (27.67)	114 (13.59)	< 0.001	132 (15.73)	114 (13.59)	0.214
Previous aortic valve disease, n (%)	483 (26.41)	206 (24.55)	0.309	192 (22.88)	206 (24.55)	0.422
Previous mitral valve disease, n (%)	402 (21.98)	268 (31.94)	< 0.001	260 (30.99)	268 (31.94)	0.674
Previous pulmonic valve disease, n (%)	2 (0.11)	2 (0.24)	0.424	1 (0.12)	2 (0.24)	0.563
Previous tricuspid valve disease, n (%)	104 (5.69)	94 (11.2)	< 0.001	73 (8.7)	94 (11.2)	0.087
Prosthetic valve carriers, n (%)	173 (9.46)	83 (9.89)	0.724	84 (10.01)	83 (9.89)	0.935
CCI index, mean (SD)	1.47 (1.13)	1.41 (0.99)	0.170	1.26 (1.05)	1.41 (0.99)	0.004
Periannular complications / atrioventricular block, n (%)	122 (6.67)	45 (5.36)	0.196	53 (6.32)	45 (5.36)	0.405
Septic arterial embolism, n (%)	67 (3.66)	35 (4.17)	0.525	34 (4.05)	35 (4.17)	0.902
Cardiogenic shock, n (%)	35 (1.91)	14 (1.67)	0.662	9 (1.07)	14 (1.67)	0.294
Dialysis, n (%)	118 (6.45)	64 (7.63)	0.263	41 (4.89)	64 (7.63)	0.020
Heart valve surgery (aortic, mitral, tricuspid, pulmonary), n (%)	336 (18.37)	121 (14.42)	0.012	123 (14.66)	121 (14.42)	0.890
Mechanical ventilation, n (%)	207 (11.32)	87 (10.37)	0.468	65 (7.75)	87 (10.37)	0.061
Pacemaker implantation, n (%)	121 (6.62)	41 (4.89)	0.083	50 (5.96)	41 (4.89)	0.332
LOHS, Median (IQR)	19 (25)	18 (25)	0.257	19 (26)	18 (25)	0.329
IHM, n (%)	302 (16.51)	190 (22.65)	< 0.001	151 (18.00)	190 (22.65)	0.018

T2DM: Type 2 diabetes mellitus; CCI: Charlson comorbidity index; COPD: chronic obstructive pulmonary disease; LOHS: Length of hospital stay; IHM: in-hospital mortality

After PSM, women with T2DM had higher mean CCI values (1.41 ± 0.99 vs. 1.26 ± 1.05; p = 0.004), and men with T2DM received dialysis significantly less often than women with T2DM (4.89% vs. 7.63%; p < 0.001). The difference in IHM was statistically significant (p = 0.018), with mortality of 18.0% for men with T2DM and 22.65% for women with T2DM.

As for the pathogen isolated, after PSM, Gram-negative bacteremia was significantly more prevalent in women with T2DM than in men with T2DM (12.16% vs. 7.27%; p = 0.001) (Additional file 1: Table S4).

### Variables associated with IHM in diabetic men and women with IE: Multivariable analysis

Multivariable adjustment showed that the IHM was associated with old age (≥ 76 years old) and the presence of acute renal disease, congestive heart failure, septic arterial embolism, and cardiogenic shock among men and women with T2DM (Table 5). Ischemic heart disease was associated with IHM in women with T2DM, but not in men.

**Table 5:** Multivariable analysis of factors associated with in-hospital mortality with infective endocarditis, among T2DM patients according to sex

	MEN	WOMEN	BOTH
	OR (95%CI)	OR (95%CI)	OR (95% CI)
40–66 years old	1	1	1
67–75 years old	1.34 (0.93–1.95)	1.13 (0.61–2.09)	1.04 (0.66–1.65)
≥ 76 years old	2.15 (1.5–3.09)	1.8 (1.01–3.25)	1.52 (0.97–2.36)
Acute renal disease	2.33 (1.75–3.11)	2.27 (1.52–3.4)	2.01 (1.52–2.67)
Congestive heart failure	1.52 (1.12–2.02)	1.78 (1.21–3.02)	1.61 (1.21–2.33)
Ischemic heart disease	NS	1.64 (1.01–2.7)	1.75 (1.19–2.58)
Septic arterial embolism	2.52 (1.14–5.57)	4.55 (2.1–9.85)	2.88 (1.86–4.66)
Cardiogenic shock	2.76 (1.25–6.1)	6.99 (1.89–25.84)	3.87 (1.48–10.12)
Dialysis	2.27 (1.43–3.61)	3.78 (2.03–7.05)	2.21 (1.38–3.54)
Mechanical ventilation	2.58 (1.74–3.81)	3.16 (1.76–5.69)	3.44 (2.26–5.23)
Pacemaker implantation	0.29 (0.14–0.61)	NS	0.44 (0.22–0.89)
Staphylococcus bacteremia	1.33 (1.01–1.78)	NS	1.47 (1.11–1.95)
Streptococcus bacteremia	0.48 (0.31–0.76)	0.57 (0.33–1)	0.49 (0.32–0.73)
Female sex	NA	NA	1.24 (1.02–1.59)

The need for mechanical ventilation during admission was associated to higher IHM in T2DM patients irrespective of sex: men, OR, 2.58 (95%CI, 1.74–3.81); women, OR, 3.16 (95%CI, 1.76–5.69). However, the need for pacemaker was associated to lower IHM only in men with T2DM (OR, 0.29; 95% CI, 0.14–0.61).

As for pathogens, *Streptococcus* bacteremia was associated with lower IHM in men and women with T2DM, as compared with T2DM patients with no positive blood cultures. Although *Staphylococcus* bacteremia was associated to a higher IHM only in men with T2DM (OR, 1.33; 95%CI, 1.01–1.78).

As found with PSM, being a women with T2DM was significantly associated to higher IHM when compared to T2DM men (OR, 1.24; 95% CI, 1.02–1.59) (Table 5).

Finally, T2DM was not associated with IHM in the whole population of patients with IE: men, OR, 0.87 (95%CI, 0.72–1.05); women, OR, 0.93 (95%CI, 0.72–1.2) (Table 6).

**Table 6:** Multivariable analysis of factors associated with in-hospital mortality with infective endocarditis, among all patients according to sex

	MEN	WOMEN	BOTH
	OR (95%CI)	OR (95%CI)	OR (95%CI)
40–66 years old	1	1	1
67–75 years old	1.34 (1.02–1.76)	1.14 (0.73–1.78)	1.35 (1.07–1.69)
≥ 76 years old	2.22 (1.71–2.89)	1.87 (1.24–2.84)	2.12 (1.7–2.64)
Acute renal disease	2.12 (1.73–2.6)	2.19 (1.65–2.9)	1.98 (1.68–2.33)
Chronic renal disease	1.22 (1.01–1.51)		1.18 (1–1.39)
Congestive heart failure	1.96 (1.72–2.42)	2.11 (1.79–3.20)	2.01 (1.68–2.87
Ischemic heart disease	1.27 (1.04–1.55)	1.41 (1.14–1.72)	1.34 (1.11–1.57)
Septic arterial embolism		3.73 (2.07–6.73)	1.78 (1.24–2.56)
Cardiogenic shock	4.35 (2.59–7.28)	5.5 (2.24–13.5)	4.02 (2.58–6.25)
Dialysis	1.8 (1.27–2.56)	3.4 (2.07–5.58)	1.91 (1.44–2.53)
Heart valve surgery (aortic, mitral, tricuspid, pulmonary)	0.74 (0.56–0.97)		
Mechanical ventilation	3.49 (2.65–4.6)	2.63 (1.73–4)	3.06 (2.44–3.84)
Pacemaker implantation	0.34 (0.2–0.57)		0.34 (0.22–0.53)
Staphylococcus bacteremia	1.29 (1.05–1.58)		1.49 (1.26–1.74)
Streptococcus bacteremia	0.44 (0.32–0.59)	0.39 (0.26–0.58)	0.43 (0.34–0.55)
Female sex	NA	NA	1.31 (1.11–1.54)
T2DM	0.87 (0.72–1.05)	0.93 (0.72–1.2)	0.92 (0.8–1.07)

T2DM: Type 2 diabetes mellitus

## Discussion

This nationwide registry and population-based observational cohort study showed that the incidence of hospitalizations for IE was higher in men and women with T2DM than in those without T2DM for all the years analyzed. No differences in IHM were found between patients with and without T2DM, although IHM was significantly higher in women with T2DM than in men with T2DM. Comorbidity, in-hospital dialysis, and mechanical ventilation were associated with higher IHM in T2DM patients. In the fully adjusted model, being a women with T2DM was associated to a 24% higher IHM after IE than men with T2DM.

### Incidence of IE according to T2DM status

In our investigation 9,958 cases of IE in subjects aged 40 years were identified over a 5-year period (2016–2020), this represents a yearly incidence of 7.60 cases per 100,000 inhabitants’ ≥ 40 years. The epidemiological and clinical characteristics of IE are known to exhibit substantial geographical variability [4, 6, 27–29]. The reported incidence of IE among different studies is not entirely similar ranging between 3 and 15 cases per 100,000 in population-based studies, with considerable differences noted even in similar countries [4, 6, 27–29]. Very recently, in a territory-wide study in Hong Kong the incidence of IE was recorded as 5.4 (95% CI 5.1 to 5.7) cases per 100,000 person-year between 2016 and 2019, and after adjustment for age and sex, the incidence did not significantly change over time [27]. In Portugal, from 2010 to 2018, the incidence of IE varied between 6.25 cases and 9.35 per 100,000 [28]. Studies conducted in other European countries and the USA have reported higher figures [4, 6, 29].

In Spain IE is an uncommon disease with low incidences when compared with other counties. Results of the Grupo Español de Endocarditis Infecciosa [Spanish Collaboration on Endocarditis] (GAMES), an observational, multicentric, prospective study based on a nationwide registry that included all consecutive patients with a diagnosis of definite IE according to the modified Duke criteria, have reported an incidence ranging from 3 to 4 cases per 100.000 inhabitants’ year [16, 30, 31].

In our population the incidence only rose among people with T2DM remaining stable among those who don’t have this condition. Trend in the incidence show conflicting results with some countries showing increments and other no changes overtime [4, 6, 27–29, 32]. Talha et al. conducted a systematic review that included population-based incidence of IE in patients, irrespective of age, in European countries. The pooled regression estimate was 4.1% ± 1.2% per year increase in IE incidence, amounting to a compound increase in incidence of 106% over 18 years (2000–2018) [32].

In Spain the increase in the general population and among those with T2DM has been reported before, even if the increment seems to be slower than in other countries [3, 8, 30, 31, 33]. The trend suggests that, as reported elsewhere, the increase could be partly explained by aging, the burden of comorbidity, and a progressively higher number of invasive procedures [2, 34]. In our study, the mean CCI and frequency of previous tricuspid and aortic valve disease increased significantly over time in patients with T2DM.

As expected, we found that incidence was higher in patients with T2DM, irrespective of sex. Cellular immunity and phagocytic function tend to be impaired in persons with diabetes, thus predisposing them to severe infections [1, 35].

In our opinion the increase overtime in the prevalence of comorbidities among patients with and without T2DM hospitalized with IE is probably due to the aging of the Spanish population and has been previously described in Spain and in other countries [3, 27–29, 31]. However, improvements in the coding practices overtime may have also contributed to the increment.

### Differences in the clinical characteristics, hospital outcomes and microorganisms according to T2DM status

The clinical profile of IE patients with T2DM differed from that of patients without T2DM (higher rates of comorbidities, and risk factors for IE such as older age, presence of a pacemaker, and in-hospital dialysis). Our findings indicate that during admission for IE, men and women with T2DM received dialysis more frequently than matched non-T2DM men and women. In a study about risk factors and outcomes of early acute kidney injury in IE, the authors concluded that IE due to history of diabetes (OR, 2.34; 95% CI, 1.25–4.37; p < 0.01) was associated with early acute kidney injury [36]. Furthermore, dialysis was more frequently used in T2DM women than in T2DM men. Rates of dialysis in women with T2DM are higher because women with IE experience more hospital complications than men, potentially affecting treatment decisions that involve a less invasive approach than dialysis. As reported elsewhere, dialysis was a risk factor for IHM in both men and women with T2DM [8].

We were not surprised to find that crude IHM in patients with and without T2DM remained stable between 2016 and 2020, probably because of recent improvements in the management and pharmacological treatment of T2DM patients in Spain [37].

The organisms responsible for IE also differed significantly. *Staphylococcus* species was more frequent in T2DM patients and proved to be a risk factor for IHM in men with T2DM. This finding is consistent with those of previous studies and likely due to increased health care utilization in T2DM patients, who are thus exposed to nosocomial infections and immune dysfunction, leaving them more susceptible to skin and soft tissue infections [7].

A remarkable finding or our study was the surprisingly high (7%) proportion of gram-negative bacteremia. Previous hospital cohorts’ studies conducted in our country have reported lower values, ranging from 4.5% to 5.5% of IE patients [19, 31, 38, 39].

However, in Portugal, using administrative data 11.9% of cultures were codified as a gram-negative bacterium [28]. Possible reasons for this high prevalence may include advancements in culturing methods and a survival bias due to the good prognosis of the HACEK group IE [39, 40]. However future investigations should assess the validity of ICD codes for microorganisms in our country.

### Sex differences in the incidence and outcomes of IE among patients with T2DM

In our study, the incidence of hospitalizations for IE was higher in T2DM men than in T2DM women. These results are consistent with the findings of previous studies on IE in the general population, which demonstrated that IE is more frequent in males [12, 13]. The aortic valve was the most frequently previously affected in men, whereas in women, the mitral valve was affected in 31.94% of cases. This finding agrees with previously published results, since the location of IE differed between the sexes because of differences in the predisposing lesions [14, 16]. The reasons for this sex-specific difference remain unclear, although a higher rate of predisposing heart conditions in men [13] may contribute to a lower incidence of IE in women. Hormonal factors are thought to protect women from endothelial damage and, therefore, lower their susceptibility to IE [41].

Many aspects of IE are similar in male and female patients, as reported by Polishchuk et al. [14]. We did not find any differences between T2DM men and women in LOHS, heart valve surgery procedures, mechanical ventilation, or presence of a pacemaker. However, regarding isolation of the pathogen, we found significantly more cases of Gram-negative bacteremia in women with T2DM than in men with T2DM. Other studies have reported similar results [19]. Gram-negative bacteremia could have its origin in urinary tract infections, which are more common among women. Surprisingly, we did not confirm findings from a previous study, in which *Staphylococcus aureus* was more often the causative microorganism in women than in men (30.1% vs. 23.1%; p < 0.001) [16]. These variations may reflect the local epidemiology of IE, diagnostic criteria, initiation of antibiotics before blood cultures, and the diagnostic protocol used to establish etiology [42].

### Variables associated with IHM among patients hospitalized with IE

The results of the present study are consistent with the literature finding that older age, acute renal disease, congestive heart failure, septic arterial embolism, and cardiogenic shock were factors associated with IHM after IE in patients with T2DM [7]. However, our multivariable analysis did not show T2DM to be associated with higher IHM. Contrasting and similar results have been published [8, 34]. Further larger, prospective, and more detailed studies are needed to clarify this issue. These should cover variables such as therapy for DM and glycemic control.

The multivariable analysis showed that female sex was an factor associated with mortality in T2DM patients with IE after controlling for the remaining variables analyzed. The few studies that have analyzed the influence of sex on the outcomes of IE in the general population reported contradictory results [14, 16–18]. In Spain, Varela-Barca et al. [16] found that IHM was 41% higher in women than in men (OR, 1.41; 95% CI 1.21–1.65). These authors indicate that since hormonal differences protect young women from cardiovascular disease, women develop heart disease later in life. Our findings reflect this observation in that the age of presentation of the IE episode differed significantly between the sexes (75.58 ± 9 years in women with T2DM and 71.61 ± 9.88 years in men with T2DM). Data reported elsewhere show that the higher mortality rate in women is associated with poorer baseline characteristics in women [16, 17]. We also found a significant difference in the mean CCI between women and men with T2DM (1.41 [SD = 0.99] and 1.26 [SD = 1.05], respectively). Finally, in Spain, this association has been linked to sex-related differences in the frequency of heart valve surgery [17, 43], although this did not differ between men and women in our study.

Is remarkable that in a previous study conducted by our group, among T2DM patients who had experienced a hemorrhagic stroke, similar sex differences were detected, finding that men presented higher incidence rates, more frequent decompressive craniectomy, and lower in-hospital mortality than women [44].

We are unable to provide a rational explanation for this finding despite its importance. Limited research has been conducted so far on women’s health with cardiovascular conditions such as IE and women have been frequently excluded from clinical trials. For these reasons, the prevention, diagnosis, and treatment of cardiovascular disease in women continue to be based on findings in men, and sex-specific clinical guidelines are mostly lacking [45]. As suggested by Regensteiner et al., sex and gender should be incorporated into the design of prospective trials to ensure that outcomes and the implementation of findings are broadly and appropriately applicable to patient care [45].

### Strengths and limitations

The strength of our study lies in its large sample size (data from over 9,958 episodes of IE, 26.79% with T2DM), the fact that it covers the population of an entire country (> 95% of all hospital admissions) and the standardized methodology (extensively used for research in IE in Spain).

Nevertheless, our work is subject to a series of limitations. First, the accuracy of the medical information included in the RAE-CMBD could not be verified at an individual patient level and therefore incomplete or erroneous information could have been included. Previous studies have assessed the validity of ICD codes for IE in administrative databases [4, 27, 29, 46–48]. Fawcett et al., reported a sensitivity of IE of 76% for specific codes in ICD-10 but more than half of cases coded by using ICD-10 as IE were not confirmed cases. The code I33 had a positive predictive value (PPV) of 82%-85%; in contrast, and the code I38 had a PPV of < 6% and accounted for many of the false-positive cases [46]. By contrast, in Canada, Tan et al. conducted a retrospective validation study of ICD-10 codes for IE against clinical Duke criteria (definite and probable) at a large acute care hospital between 2013 and 2015 finding that the ICD-10 codes had a sensitivity of 90% (95% CI 81–95), specificity of 100% (95% CI, 100–100), PPV of 78% (95% CI; 68–85) and negative predictive value of 100% (95% CI, 100–100) [47]. Concluding that that the ICD-10-CM codes for IE have strong diagnostic accuracy. However, this study is limited by a relatively small sample size from a single center [47].

In Scotland electronic hospital records of 396 episodes of suspected IE codified with ICD9 and ICD10 dating from a 5-year period (2014–2018) were manually reviewed [4]. Reporting that when IE appeared in any diagnostic code position the PPV was 67.9% (95% CI 64.0–71.8) [4]. In Taiwan among 593 adult patients with discharge ICD codes for IE (ICD-9: and ICD-10:) during 2005–2016 in a single-center setting only 57% met the modified Duke criteria (PPV 57%, 95% CI 53–61) after systematically reviewed the medical charts [48].

However, studies conducted in single centers in the USA and China using ICD 9 have reported much better results [27, 29]. Toyoda et al. identified 283 patients with any type of IE, finding a sensitivity, specificity, and PPV of the ICD-9-CM codes, for acute IE defined by the modified Duke criteria, of 94% (95% CI, 92%-97%), 99% (95% CI, 99%-99%), and 94% (95% CI, 91%-97%) respectively [29]. Li et al., in their validation exercise, there was a high diagnostic accuracy with a PPV of 88.8% (95% CI 84.8 to 92.9) [27].

Inaccurate coding may contribute to a moderate PPV and may be caused by clinicians' inexperience or attention to detail. Poor specificity of coding data could be explained by several coding erroneous practices, such as a readmission or historical cases with hospitalizations that did not address the endocarditis as a current problem. Moreover, the accuracy of ICD-based phenotyping can be affected by variations in the policies and regulations of a health insurance system, the population covered by the healthcare system, and the coding behavior of clinicians, which consequently affect the interpretation and validity of clinical research findings [46–48]. In Spain, as in most countries, trained health record coders enter administrative codes according to standardized protocols; however, our results may not be generalizable to countries in which entry is performed by untrained medical staff.

Several authors have suggested that the cumulative incidence of IE may be overestimated when ICD codes are used to identify IE patients, but the mortality of IE be underestimated [46, 48].

The prevalence of diabetes (26–29%) and the sex distribution (66–68% men) found in the GAMES registry are very similar to the corresponding values found in our study population (26.79% T2DM and 66.79% men) and this suggests the validity of our results [16, 31, 34]. In any case, in our opinion, there is no reason to think that coding validity differs among patients with and without T2DM. Therefore, indicating that misclassification bias would be non-differential, and this could result in reducing the magnitude, towards the null, but not changing the direction of any possible associations [49].

Second as our data source was an administrative database supported by the information that physicians recorded in discharge reports; therefore, we lack information on clinical characteristics, glycemic control, medical treatments, and the duration of T2DM. Third, the only data available were those included in the ICD-10 coding on duration of ventilatory support, days in intensive care, and duration of dialysis. Fourth, while PSM helped to attenuate differences in baseline characteristics and clinical variables, it is difficult to eliminate residual confounding in observational studies. Fifth, our study is also limited by the fact that causative pathogens were only identified around 60%. Shah et al., in Scotland using hospital discharge codes, reported that, even with linkage to a robust national microbiology laboratory blood culture dataset, the causative organism was not identified in the majority (57%) of patients with IE [4] In Hong Kong from 2002 to 2019 the rate of culture-negative endocarditis was 35.4% [27]. In Portugal for the period 2010–2018 with a methodology very similar to ours a microorganism was coded only in 49.5% of the incident episodes of IE [28]. Better results were obtained by Toyoda et al. in the USA who reported that among the entire cohort, 75% of patients had a causative microorganism coded [29]. Another limitation of ICD codes-based investigations is that the organisms identified were assumed to be causative if they were coded during the hospitalizations with IE, but this could not be validated for individual patients. It was also not possible to differentiate between culture-negative IE and IE for which the causative organism was simply not recorded or done.

In Spain the results of hospital-based cohorts show much lower proportions of culture-negative endocarditis with figures ranging 10–20% [31, 34, 50]. In a recent study conducted among 3113 IE patients admitted to hospitals in Europe, from January 2016 to March 2019, a positive culture was obtained in 83.2%, whereas 16.8% had a culture-negative [51]. However, this very high rates are expected because in most hospitalized cohorts of patients with IE, cases were identified by the attending clinician and a high culture-positive rate in these cohorts may therefore reflect selection bias toward patients with positive blood cultures [4].

Cultures are negative in IE for three major reasons: previous administration of antimicrobial agents, inadequate microbiological techniques, infection with highly fastidious bacteria or nonbacterial pathogens [4, 50, 51].

Sixth, in our study we considered a patient to have T2DM if a code (IC10 E11.x) was recorded in any diagnosis field beside it the “*Present on Admission*” (POA) indicator was “*Yes*” or “*No*”. This last option means that T2DM was diagnosed during the hospitalization. However, in our study population very few patients had T2DM diagnosed when admitted with IE (< 1%), this agrees with studies conducted in other countries [52]. Seventh, in Spain the use of outpatient parenteral antibiotic treatment (OPAT) for IE has been implemented in some hospitals, even if the proportion of patient who undergo this therapy is low [53]. However, in all cases the initial treatment is provided as in-patients [53]. Unfortunately, with the RAE-CMBD we cannot distinguish patients with OPAT so the effect of this type of treatment could not be assessed. Finally, as we conducted an observational study we could establish relations between variables, but not confirm causality.

## Conclusions

In conclusion, rates of hospitalization for IE only increased in men with T2DM during the period 2016–2020 and with significantly higher incidence rates in T2DM patients. Our population-based study showed that T2DM was not a predictor of IHM after IE in men and women. Our data showed major sex differences, indicating that female sex is a predictor of IHM in IE in persons with T2DM. Older age, comorbidities, dialysis, and mechanical ventilation were associated with higher IHM in men and women with T2DM and IE, whereas presence of a pacemaker predicted lower IHM only in men with T2DM. However, given the limitations of the RAE-CMBD, our conclusions must be corroborated by prospective studies including detailed clinical data.

## Supplementary Information

**Additional file 1: Table S1.**. Number of men and women aged 40 years or over discharged from Spanish hospitals from year 2016 to year 2020. Data collected by the Hospital Discharge Records of the Spanish National Health System (RAE-CMBD, Registro de Actividad de Atención Especializada-Conjunto Mínimo Básico de Datos (RAE-CMBD). **Table S2.** Diagnosis, procedures and pathogens analyzed with their corresponding ICD10 codes. **Table S3.** Distribution of pathogens in patients with and without T2DM with infective endocarditis in Spain from 2016 to 2020. **Table S4.** Distribution of pathogens in women and men with T2DM with infective endocarditis, in Spain (2016-2020), before and after propensity score matching.

## Data Availability

According to the contract signed with the Spanish Ministry of Health and Social Services, which provided access to the databases from the RAE-CMBD: *Registro de Actividad de Atención Especializada-Conjunto Mínimo Básico de Datos* (Register of Specialized Care–Basic Minimum Database), we cannot share the databases with any other investigator, and we have to destroy the databases once the investigation has concluded. Consequently, we cannot upload the databases to any public repository. However, any investigator can apply for access to the databases by filling out the questionnaire available at http://www.msssi.gob.es/estadEstudios/estadisticas/estadisticas/estMinisterio/SolicitudCMBDdocs/Formulario_Peticion_Datos_CMBD.pdf. All other relevant data are included in the paper.

## Abbreviations

CCI: Charlson Comorbidity Index
CI: Confidence interval
GAMES: Grupo Español de Endocarditis Infecciosa [Spanish Collaboration on Endocarditis]
HR: Hazard ratio
ICD-10: International Classification of Disease, Tenth Revision
IE: Infective endocarditis
IHM: In-hospital mortality
IQR: Interquartile range
IRR: Incidence rate ratio
LOHS: Length of hospital stay
OPAT: Outpatient parenteral antibiotic treatment
ORs: Odds ratios
PPV: Positive predictive value
PSM: Propensity score matching
RAE-CMBD: *Registro de Actividad de Atención Especializada-Conjunto Mínimo Básico de Datos* (Register of Specialized Care–Basic Minimum Database)
SD: Standard deviation
T2DM: Type 2 diabetes mellitus
