# Supplementary material for: Sex-related disparities in the incidence and outcomes of infective endocarditis according to type 2 diabetes mellitus status in Spain, 2016–2020
Source: Cardiovasc Diabetol. 2022 Sep 30;21:198. doi: 10.1186/s12933-022-01633-2 (PMC9524731; doi:10.1186/s12933-022-01633-2)
Supplement: Supplementary file 1 — Additional file 1: Table S1.. Number of men and women aged 40 years or over discharged from Spanish hospitals from year 2016 to year 2020. Data collected by the Hospital Discharge Records of the Spanish National Health System (RAE-CMBD, Registro de Actividad de Atención Especializada-Conjunto Mínimo Básico de Datos (RAE-CMBD). Table S2. Diagnosis, procedures and pathogens analyzed with their corresponding ICD10 codes. Table S3. Distribution of pathogens in patients with and without T2DM with infective endocarditis in Spain from 2016 to 2020. Table S4. Distribution of pathogens in women and men with T2DM with infective endocarditis, in Spain (2016-2020), before and after propensity score matching. [file 12933_2022_1633_MOESM1_ESM.docx]

**Table S1.** Number of men and women aged 40 years or over discharged from Spanish hospitals from year 2016 to year 2020. Data collected by the Hospital Discharge Records of the Spanish National Health System (RAE-CMBD, Registro de Actividad de Atención Especializada-Conjunto Mínimo Básico de Datos (RAE-CMBD)

|  | **Men** | | **Women** | | **Both sexes** |
| --- | --- | --- | --- | --- | --- |
| Year | N | % | N | % | N |
| 2016 | 1,665,033 | 52.20 | 1,524,470 | 47.80 | 3,189,503 |
| 2017 | 1,743,526 | 52.10 | 1,603,090 | 47.90 | 3,346,616 |
| 2018 | 1,761,883 | 52.30 | 1,606,737 | 47.70 | 3,368,620 |
| 2019 | 1,779,752 | 52.28 | 1,624,783 | 47.72 | 3,404,535 |
| 2020 | 1,614,204 | 52.97 | 1,433,398 | 47.03 | 3,047,602 |
| All years | 8,564,398 | 52.36 | 7,792,478 | 47.64 | 16,356,876 |

**Table S2.** Diagnosis, procedures and pathogens analyzed with their corresponding ICD10 codes.

| **Diagnosis/Procedures/Pathogens** | **ICD-10 codes** |
| --- | --- |
| Previous mitral valve disease | I050; I051; I052; I058; I059; I080; I081; I083; I340; I341; I342; I348; I349 |
| Previous aortic valve disease | I060; I061; I062; I068; I069; I080; I350; I351; I352; I358; I359; I082; I083 |
| Previous tricuspid valve disease | I070; I071; I072; I078; I079; I081; I082; I083; I360; I361; I362; I368; I369 |
| Previous pulmonary valve disease | I370; I371; I372; I378; I379 |
| COVID-19 | B34.2; B97.29; U07.1 |
| Atrial fibrillation | I48.0; I48.1; I48.2; I48.91 |
| Ischemic heart disease | I20-I25 |
| Periannular complications/atrioventricular block | I51.1; I51.2; I44.2; I44.1 |
| Septic arterial embolism | I76 |
| Shock | R57.0 |
| Prosthetic valve carriers | Z95.2 |
| Dialysis | 5A1D xxx |
| Heart valve surgery (aortic, mitral, tricuspid, pulmonary) | 024F07J; 024F08J; 024F0JJ; 024F0KJ; 02BF0ZX; 02BF0ZZ; 02CF0ZZ; 02NF0ZZ; 02QF0ZJ; 02QF0ZZ; 02RF07Z; 02RF08Z; 02RF0JZ; 02RF0KZ; 02UF07J; 02UF07Z; 02UF08J; 02UF08Z; 02UF0JJ; 02UF0JZ; 02UF0KJ; 02UF0KZ; 02WF07Z; 02WF08Z; 02WF0JZ; 02WF0KZ; X2RF032  024G072; 024G082; 024G0J2; 024G0K2; 02BG0ZX; 02BG0ZZ; 02CG0ZZ; 02NG0ZZ; 02QG0ZE; 02QG0ZZ; 02RG07Z; 02RG08Z; 02RG0JZ; 02RG0KZ; 02UG07E; 02UG07Z; 02UG08E; 02UG08Z; 02UG0JE; 02UG0JZ; 02UG0KE; 02UG0KZ; 02VG0ZZ; 02WG07Z; 02WG08Z; 02WG0JZ; 02WG0KZ  024J072; 024J082; 024J0J2; 024J0K2; 02BJ0ZX; 02BJ0ZZ; 02CJ0ZZ; 02NJ0ZZ; 02QJ0ZG; 02QJ0ZZ; 02RJ07Z; 02RJ08Z; 02RJ0JZ; 02RJ0KZ; 02UJ07G; 02UJ07Z; 02UJ08G; 02UJ08Z; 02UJ0JG; 02UJ0JZ; 02UJ0KG; 02UJ0KZ; 02WJ07Z; 02WJ08Z; 02WJ0JZ; 02WJ0KZ  02BH0ZX; 02BH0ZZ; 02CH0ZZ; 02NH0ZZ; 02QH0ZZ; 02RH07Z; 02RH08Z; 02RH0JZ; 02RH0KZ; 02TH0ZZ; 02UH07Z; 02UH08Z; 02UH0JZ; 02UH0KZ; 02WH07Z; 02WH08Z; 02WH0JZ; 02WH0KZ |
| Mechanical ventilation | 5A09357; 5A09457; 5A09557; 5A1945Z; 5A1955Z; 5A1935Z |
| Pacemaker implantation | 02HKxxx; 02HLxxx; 02H4xxx; 02H6xxx; 02H7xxx; 02HNxxx; 0JH6xxx; 0JH8xxx |
| *Stapylococcus* bacteremia | A4101; A4102; A411; A412; A4901; A4902; B9561; B9562; B957; B958 |
| *Streptococcus* bacteremia | A400; A401; A403; A408; A409; A491; B950; B951; B953; B954; B955 |
| Gram-negative bacteremia | A413; A4150; A4151; A4152; A4153; A4159; B961; B9620; B9621; B9622; B9623; B9629; B963; B964; B965 |
| Fungemia | B376; B377; B409; B393; B394; B395; B399; B449 |

The ICD 10 codes for conditions included in the Charlson Comorbidity Index can be found in references:

Sundararajan V, Henderson T, Perry C, Muggivan A, Quan H, Ghali WA. New ICD-10 version of the Charlson comorbidity index predicted in-hospital mortality. J. Clin. Epidemiol 2004;57:1288–94. doi: 10.1016/j.jclinepi.2004.03.012.

Quan H, Sundararajan V, Halfon P, Fong A, Burnand B, Luthi JC, et al. Coding algorithms for defining comorbidities in ICD-9-CM and ICD-10 administrative data. Med Care. 2005;43:1130-9. doi: 10.1097/01.mlr.0000182534.19832.83.

**Table. S3.** Distribution of pathogens in patients with and without T2DM with infective endocarditis in Spain from 2016 to 2020

|  |  | **2016** | **2017** | **2018** | **2019** | **2020** | **p-value** |
| --- | --- | --- | --- | --- | --- | --- | --- |
| Fungemia, n(%) | **T2DM** | 5(1.07) | 2(0.37) | 3(0.56) | 3(0.49) | 3(0.58) | 0.676 |
|  | **No T2DM** | 7(0.51) | 3(0.21) | 5(0.31) | 10(0.66) | 7(0.51) | 0.371 |
| Gram-negative bacteremia, n(%) | **T2DM** | 33(7.05) | 53(9.93) | 52(9.67) | 62(10.08) | 38(7.41) | 0.239 |
|  | **No T2DM** | 110(7.94) | 91(6.45) | 112(6.93) | 127(8.37) | 121(8.89) | 0.084 |
| Staphylococcus bacteremia, n (%) | **T2DM** | 158(33.76) | 146(27.34) | 164(30.48) | 237(38.54) | 167(32.55) | 0.001 |
|  | **No T2DM** | 336(24.26) | 392(27.78) | 438(27.12) | 436(28.72) | 413(30.35) | 0.007 |
| Streptococcus bacteremia, n(%) | **T2DM** | 82(17.52) | 97(18.16) | 118(21.93) | 121(19.67) | 94(18.32) | 0.386 |
|  | **No T2DM** | 342(24.69) | 319(22.61) | 420(26.01) | 346(22.79) | 334(24.54) | 0.151 |

T2DM: Type 2 diabetes mellitus;

**Table. S4.** Distribution of pathogens in women and men with T2DM with infective endocarditis, in Spain (2016-2020), before and after propensity score matching.

|  | **Before PSM** | | | **After PSM** | | | |
| --- | --- | --- | --- | --- | --- | --- | --- |
|  | **T2DM Men** | **T2DM Women** | **p-value** | **T2DM Men** | **T2DM Women** | **p-value** |  |
| Fungemia, n(%) | 11(0.6) | 5(0.6) | 0.986 | 5(0.6) | 5(0.6) | 0.999 |  |
| Gram-negative bacteremia, n(%) | 136(7.44) | 102(12.16) | <0.001 | 61(7.27) | 102(12.16) | 0.001 |  |
| Staphylococcus bacteremia, n (%) | 616(33.68) | 256(30.51) | 0.105 | 269(32.06) | 256(30.51) | 0.494 |  |
| Streptococcus bacteremia, n(%) | 367(20.07) | 145(17.28) | 0.090 | 172(20.5) | 145(17.28) | 0.092 |  |
